# First insights into the nature and evolution of antisense transcription in nematodes

**DOI:** 10.1186/s12862-016-0740-y

**Published:** 2016-08-22

**Authors:** Christian Rödelsperger, Kevin Menden, Vahan Serobyan, Hanh Witte, Praveen Baskaran

**Affiliations:** 1 Department for Evolutionary Biology, Max Planck Institute for Developmental Biology, Spemannstr. 35, Tübingen, 72076 Germany; 2 Eberhard Karls University, Tübingen, Germany

**Keywords:** Conserved synteny, Non-coding RNA, Orthology, Long introns, Strand-specific, Regulatory element

## Abstract

**Background:**

The development of multicellular organisms is coordinated by various gene regulatory mechanisms that ensure correct spatio-temporal patterns of gene expression. Recently, the role of antisense transcription in gene regulation has moved into focus of research. To characterize genome-wide patterns of antisense transcription and to study their evolutionary conservation, we sequenced a strand-specific RNA-seq library of the nematode *Pristionchus pacificus*.

**Results:**

We identified 1112 antisense configurations of which the largest group represents 465 antisense transcripts (ASTs) that are fully embedded in introns of their host genes. We find that most ASTs show homology to protein-coding genes and are overrepresented in proteomic data. Together with the finding, that expression levels of ASTs and host genes are uncorrelated, this indicates that most ASTs in *P. pacificus* do not represent non-coding RNAs and do not exhibit regulatory functions on their host genes. We studied the evolution of antisense gene pairs across 20 nematode genomes, showing that the majority of pairs is lineage-specific and even the highly conserved *vps-4*, *ddx-27*, and *sel-2* loci show abundant structural changes including duplications, deletions, intron gains and loss of antisense transcription. In contrast, host genes in general, are remarkably conserved and encode exceptionally long introns leading to unusually large blocks of conserved synteny.

**Conclusions:**

Our study has shown that in *P. pacificus* antisense transcription as such does not define non-coding RNAs but is rather a feature of highly conserved genes with long introns. We hypothesize that the presence of regulatory elements imposes evolutionary constraint on the intron length, but simultaneously, their large size makes them a likely target for translocation of genomic elements including protein-coding genes that eventually end up as ASTs.

## Background

Eukaryotic genomes are packed with regulatory arsenals ranging from transcription factors, histone and DNA modifications, enhancers and silencers, to microRNAs and many other non-coding RNA species. Numerous findings that small alterations in any of these mechanisms can cause rather dramatic developmental defects [1–4], support the idea that these complex regulatory mechanisms ensure the correct spatio-temporal expression patterns that are needed to convert a fertilized egg to a fully grown specimen of its kind. Recently, another species of RNAs, so called antisense transcripts (ASTs), came into focus of research in gene regulation ([5, 6], see [7] for review). Antisense transcripts are RNA molecules that are transcribed from the opposite strand of a protein-coding gene. While many of reported ASTs are non-coding RNAs, they can also have protein-coding capacity. Several mechanisms have been discovered by which ASTs can affect the transcription of their sense counterpart in practically every transcriptional state. The transcription initiation of genes can be affected by antisense transcripts through modification of methylation states [8], recruitment of Polycomb proteins [9] or even histone modifications [10]. Antisense transcription can also affect genes co-transcriptionally by e.g. polymerase collision, which can occur when genes on opposite strands are transcribed simultaneously [11]. Isoform production of a sense mRNA can furthermore be influenced by ASTs if they bind to specific splice sites [12]. Even post-transcriptional effects can be observed as ASTs can stabilize mRNAs by binding to specific sites of the mRNA that would otherwise lead to degradation [13].

In this study, we characterize genome-wide patterns of antisense transcription in the nematode *Pristionchus pacifcus* and investigate their evolutionary conservation. *P. pacificus* has been established as a satellite model to *Caenorhabditis elegans* for comparative studies of various aspects of evolutionary biology including developmental plasticity [14, 15], immunity [16, 17], and population genomics [18, 19]. In contrast to many other studies, that explicitly aimed to identify and study non-coding RNAs [20–22], our focus is on describing antisense transcription in general, irrespective of protein-coding or non-coding potential. We sequenced a strand-specific RNA-seq library to identify ASTs and to characterize their corresponding host genes. We show that in contrast to the prominent role ASTs play in gene regulation [7], most identified ASTs in *P. pacificus* are protein-coding and do not seem to have any regulatory function with respect to their host gene. Nevertheless, we find antisense gene pairs that seem to be remarkably conserved across the nematode phylum. However, even in these highly conserved cases, we see abundant structural changes ranging from intron gain to loss of ASTs. In summary, there seems to be no obvious functional relationship beetwen ASTs and their host genes in nematodes. Antisense transcription rather appears to be a feature of highly conserved genes with exceptionally long introns. What imposes the evolutionary constraint on these long introns can only be speculated and will be subject to further studies.

## Results

### Identification of antisense transcripts in *P. pacificus*

**Fig. 1:**
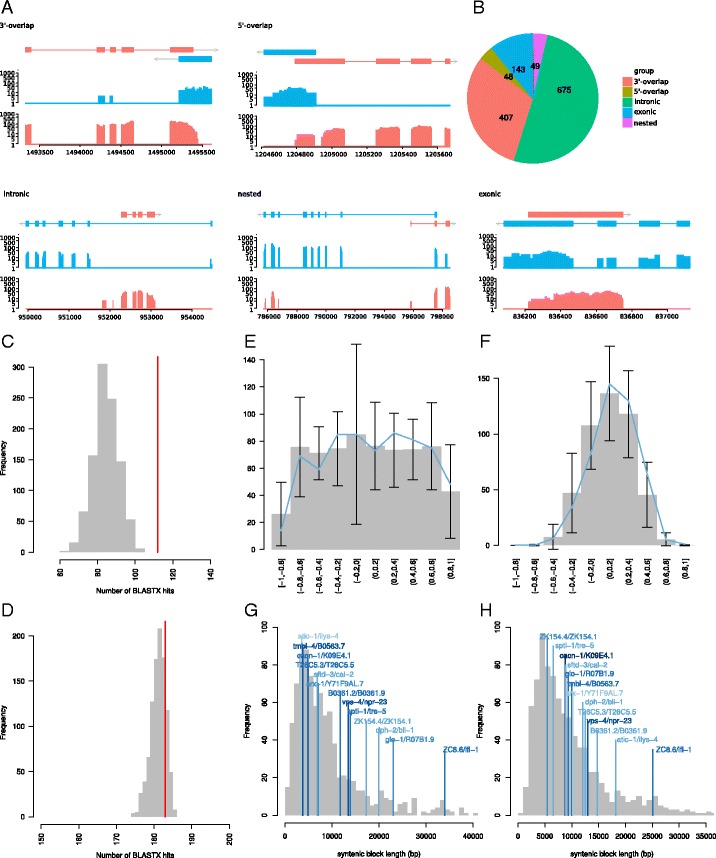
Antisense transcription in *P. pacificus* and *C. elegans*. **a** Examples of different antisense overlaps. The plots show assembled transcripts for sense and antisense transcripts and the coverage profiles on logarithmic scale. **b** Distribution of different classes of antisense overlaps. Intronic ASTs represent by far the most abundant class. **c** Simulated and observed number of BLASTX hits against available proteomic data. As our transcriptome data has directional information, we filtered out BLASTX hits that referred to the reverse strand of the query transcripts. ASTs (*red* line) show more BLASTX hits in proteomic data than expected **d** Simulated and observed number of BLASTX hits against predicted genes in *P. pacificus*. **e** Correlation between expression of ASTs and their host genes in developmental transcriptomes of *P. pacificus*. Under the assumption that ASTs could have a regulatory effect on their host genes, a subset of antisense gene pairs (*blue* lines) should show a trend towards negative or positive correlation. However, the expression correlation of antisense gene pairs is within the range of randomly chosen gene pairs suggestion no regulalatory effect. **f** Correlation between expression of ASTs and their host genes in 50 transcriptomic samples of *C. elegans*. **g** Size distribution of conserved syntenic blocks between *C. elegans* and *P. pacificus* and sizes of conserved antisense gene pairs. In *C. elegans*, conserved antisense gene pairs tend to be larger than other conserved syntenic blocks (*P*=0.03, Wilcoxon-test). **h** Size distribution of conserved syntenic blocks and antisense gene pairs in *P. pacificus*

We sequenced a strand-specific RNA-seq library of mixed-stage *P. pacificus* worm cultures resulting in 30.6 million paired end reads (2 ×101 bp). We used this data to assemble 42,821 strand-specific transcripts and tested for overlap between transcripts from different strands (see “Methods”). Figure 1a, b shows the distribution and examples of various types of overlaps between transcripts from different strands. In total, we identified 1112 antisense overlaps in *P. pacificus*. 407 of these could be classified as 3’-UTR overlap and 48 as 5’-UTR overlap. These genes overlap with their UTRs but do not share any protein-coding parts. 143 transcript pairs have overlapping protein-coding exons and are thus classified as ‘exonic overlap’. We furthermore classified all those transcripts as ’nested’ which have some exons fully embedded in antisense introns, without the whole gene being embedded in the respective intron. For the latter class, we could identify 49 transcript pairs. The largest class consists of transcripts that are completely embedded in antisense introns. For this class, we could identify 465 antisense transcripts (ASTs) that fall into 278 host genes, totalling 675 different antisense gene pairs. Thus, some host genes harbor multiple ASTs, yet closer examination of these cases showed that some ASTs sharing the same host genes show homology to the same *C. elegans* protein, thereby indicating that they were either misannotated as two genes or were recently duplicated. Since the class of antisense transcripts with shared 3’-UTR has been extensively characterized in *C. elegans* [23], we will from now on focus on intronic ASTs as they represent by far the most abundant class of ASTs. In the following, we use the term antisense gene pair to denote a combination of an AST that is fully embedded within a single intron of its respective host gene.

### Majority of antisense transcripts are protein-coding

We first tested if ASTs show any signal for non-coding RNAs. We therefore hypothesized that they should be depleted from homology to any protein-coding gene or proteomic data [24, 25]. To test this, we compared the amount of protein evidence for antisense transcripts with sets of randomly chosen transcripts having the same length distribution. In contrast to our expectation that ASTs should be depleted in protein evidence, they actually exhibitted elevated levels (compared to randomly chosen transcripts) of BLASTX hits in correct orientation against available proteomic data (Fig. 1c) and predicted protein-coding genes (Fig. 1d). The different protein-coding potential between ASTs and randomly chosen transcripts is probably due to differences in proportions of non-coding RNAs, protein-coding genes, but also mis-annotated transcripts. However, the main conclusion from this analysis is that ASTs do not show a significant depletion of protein evidence suggesting that they represent to a large extend protein-coding genes. Consequently, this indicates that antisense transcription in general is a poor predictor of non-coding RNAs. Consistently, a previous study that screened for non-coding RNAs in *C. elegans* identified only 60 candidates for antisense non-coding RNAs [20]. As ASTs tend to be protein-coding, we used the annotation of protein-coding genes from *C. elegans* to identify an analogous set of 467 ASTs embedded in 362 host genes in the *C. elegans* genome for further analysis.

### Uncorrelated expression profiles between ASTs and host genes

Even though most ASTs do not seem to represent non-coding RNAs, we tested whether ASTs could have any regulatory impact onto their host genes or vice versa. As antisense transcription has been reported to affect gene expression by polymerase collision [11], we tested whether gene pairs of host genes and their ASTs are more likely to show correlated or anticorrelated expression than random gene pairs. We obtained developmental expression profiles from *P. pacificus* [26] and large-scale expression profiles for *C. elegans* (see “Methods”), and compared the distribution of Spearman correlation coefficients between expression levels of antisense gene pairs. Based on this analysis, we find that antisense gene pairs do not show any non-random signal towards correlated nor anticorrelated expression (Fig. 1e, f). This suggests that expression of ASTs and host genes are controlled by independent regulatory programs with no evidence of interference, which is in contrast to studies in mammals where coexpression of antisense gene pairs has been found [5, 6].

### Conserved gene pairs represent unusually large blocks of conserved synteny

To assess the degree of conservation of antisense gene pairs, we used TBLASTN searches and orthology predictions between *P. pacificus* and *C. elegans* [26] to test whether orthologous host genes harbor the same antisense transcripts in both species. Indeed, we initially found 13 cases where antisense gene pairs are clearly conserved between *C. elegans* and *P. pacificus* (Fig. 1g, h). In addition, comparing the size of the spanned conserved syntenic blocks with all syntenic blocks between *C. elegans* and *P. pacificus*, we find that antisense gene pairs in *C. elegans* span rather large genomic regions as compared to conserved syntenic blocks in general (*P*=0.03, Wilcoxon test). A similar trend can also be observed in *P. pacificus*, yet the comparison is not significant (*P*=0.11, Wilcoxon test). As some conserved gene pairs were probably missed due to the incomplete assembly of host genes from poly-A enriched RNA-seq data, the statistical power would therefore be increased with more complete transcriptome data. The observation of unusually large blocks of conserved synteny does not imply that antisense gene pairs are in any way more conserved than expected, as conservation on the integrity of the host gene alone coupled with a larger gene size (see below) could result in the same pattern.

### Limited conservation of antisense transcription in nematodes

**Fig. 2:**
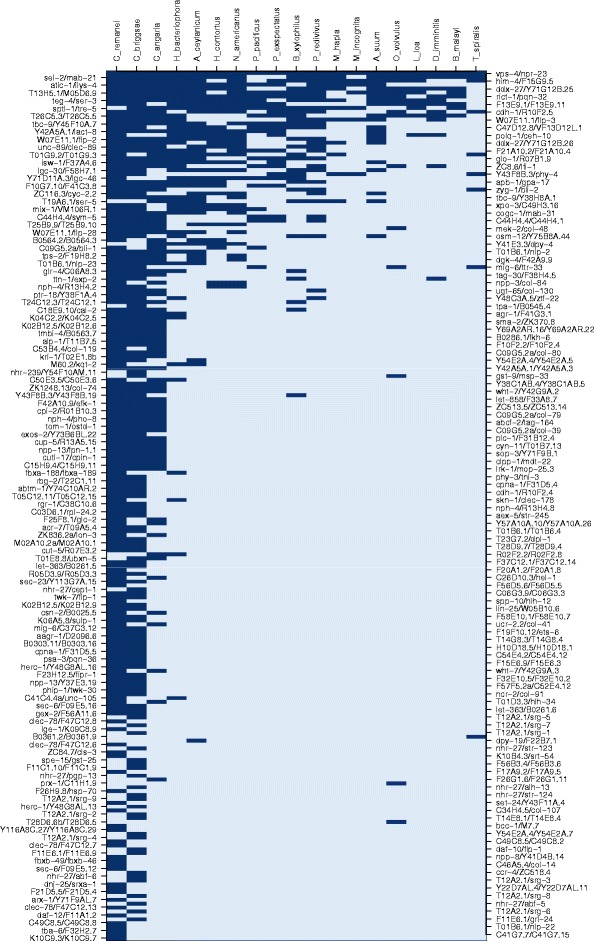
Little conservation of antisense transcripts across nematodes. Combinations of host genes and antisense transcripts from *C. elegans* were searched against 19 nematode genomes. If TBLASTN hits of the host gene span hits for the antisense transcripts on the opposite strand, a gene pair was marked as potentially conserved (*dark blue*). While spurious alignments and assembly problems can generate individual false negative and false positive calls, the overall distribution of candidates for conservation robustly indicates a strong lineage-specific signature, showing that even in the genus *Caenorhabditis* a large number of associations between host genes and antisense transcripts has either been lost or was specifically gained in the *C. elegans* lineage

**Fig. 3:**
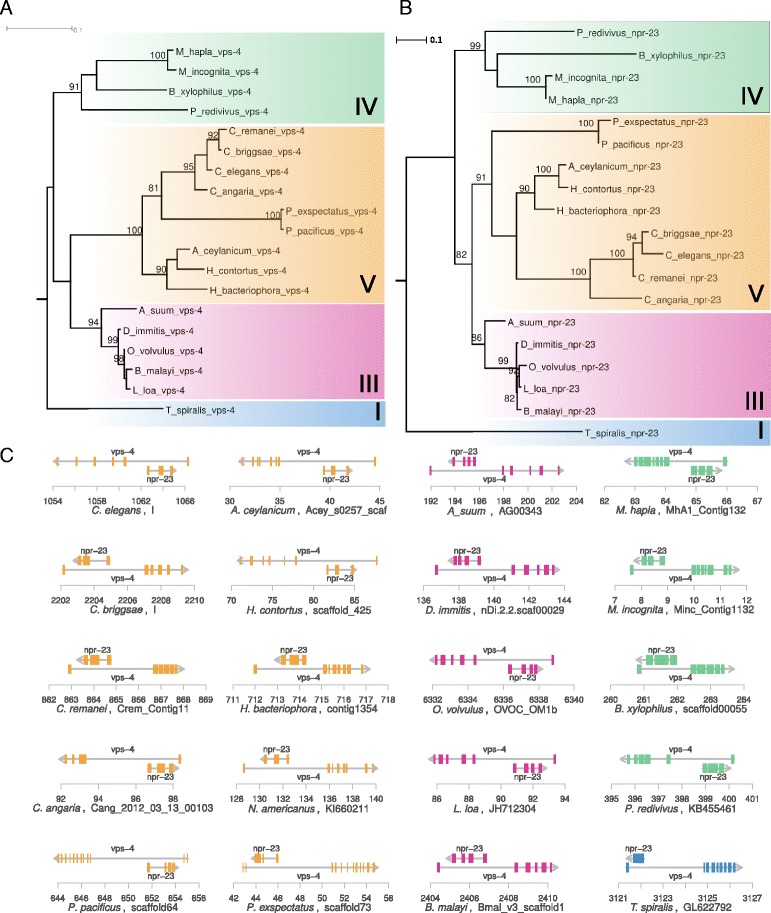
Gain of antisense introns in the highly conserved *vps-4* locus. **a** Maximum-likelihood tree of *vps-4* as retrieved by aligning the *C. elegans* *vps-4* against 19 nematode genomes. The phylogeny of *vps-4* orthologs is in agreement with the species tree, i.e. nematode species clades (I, III-V) appear in individual subtrees, indicating that the identified sequences represent one-to-one orthologs of *vps-4*. **b** Gene tree of *npr-23*. **c** genomic configuration of the *vps-4* and *npr-23* in 20 nematode genomes. This gene pair represents the only case of conservation across the complete nematode phylum. Nevertheless, this example shows that *npr-23* has gained introns independently in clade III nematodes as well as in the *Pristionchus* lineage. Please note that *vps-4* has three additional 5’exons in *C. elegans* which are not visible here as only conserved parts of the *vps-4* gene structure are shown

As RNA-seq data may not be sufficient to identify either host genes or ASTs in case that they are only lowly expressed, we used additional TBLASTN searches to screen for further candidates of conserved antisense gene pairs between *C. elegans* and *P. pacificus*. Furthermore, in order to get an overview of the global degree of conservation of antisense gene pairs in the phylum Nematoda, we performed the same search in 18 additional nematode genomes taking the *C. elegans* gene pairs as queries (Fig. 2). While most antisense gene pairs are restricted to a small set of *Caenorhabditis* species, we find a small number of gene pairs that shows broad conservation across the nematode phylum. The pair *vps-4*/*npr-23* is even predicted to be conserved in all 20 nematode genomes (Figs. 2 and 3). *vps-4* encodes an ATPase for which known functions in *C. elegans* include dissociation of the endosomal sorting complexes required for transport (ESCRT) from the endosomal membrane, and mutation of the *C. elegansvps-4* gene have been shown to lead to larval lethality [27], enlarged endosomes [28] and can even lead to abnormal distribution of embryos in the uterus [29]. *npr-23* is a G protein-coupled receptor, which is a family of genes that is proposed to play important roles in chemosensation in *C. elegans* [30]. To gain further information about the conservation of antisense gene pairs, we reannotated the corresponding genomic regions for a few highly conserved gene pairs and investigated conserved and divergent features in greater detail.

### Gene structure variation in the highly conserved *vps-4*/*npr-23* pair

We mapped the *C. elegans* protein sequences of the most highly conserved pair *vps-4*/*npr-23* against 19 nematode genomes and reconstructed gene trees for both genes (Fig. 3). As expected, phylogenetic reconstructions of both gene trees (Fig. 3a, b) show good agreement with the species tree and major nematode clades are robustly separated [31, 32]. Together with the synteny information (Fig. 3c) and the fact that BLASTP searches against the NCBI nr database could not identify any inparalogs, this confirms orthology for *vps-4* and *npr-23* across all analyzed genomes. At the level of gene structures a number of conserved as well as divergent patterns are visible. *npr-23* resides in all species in the first conserved intron of *vps-4*, which happens to be the largest intron. While the relative position of *npr-23* within this intron of *vps-4* is highly conserved, the gene structure of *npr-23* shows independent gains of introns in the lineages leading to clade IV nematodes and to the *Pristionchus* lineage (Fig. 3c).

### Gene gain and loss in the *ddx-27* locus

**Fig. 4:**
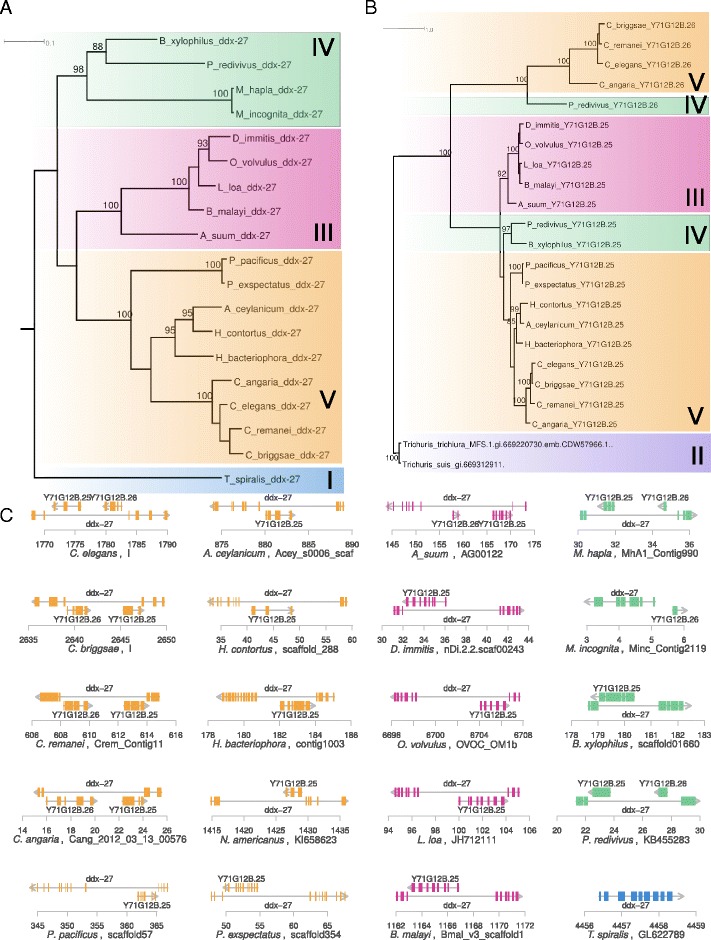
Duplication and gene loss at the *ddx-27* locus. **a** Gene tree of *ddx-27*. *ddx-27* is conserved across all analyzed nematode genomes. **b** Gene tree of Y71G1G12B.25 and Y71G1G12B.26. **c** Genomic configuration of host gene and ASTs in 20 nematode genomes. With exception of *T. spiralis*, it has up to two ASTs that have most likely arisen through a duplication event in the ancestor of clade IV and V nematodes. Subsequently, one of the copies was lost independently in various lineages. The *ddx-27* locus in *M. incognita* was split between Minc_Contig329 and Minc_Contig2119. The genes Y71G1G12B.25 and Y71G1G12B.26 could be identified in both *Meloidogyne* species but had large gaps and were therefore not included in the phylogenetic analysis

In a second example, we investigated the highly conserved *ddx-27* locus, which encodes an RNA helicase that belongs to the DEAD/H box protein family and has been characterized as essential in *C. elegans* [33]. In *C. elegans*, the *ddx-27* locus harbors two ASTs Y71G12B.25 and Y71G12B.26, which both encode Major facilitator superfamily proteins. While *ddx-27* is present in all analyzed nematode genomes (Fig. 4a), the ASTs are restricted to certain nematode lineages (Fig. 4b, c). We interpret these relationships as evidence that the AST was duplicated in the ancestor of clade IV and V nematodes and was lost independently in different nematode lineages. Alternatively the AST could have been duplicated independently in clade IV and V. However, it seems extremely unlikely that independent duplication events should result in paralogs that cluster together in phylogenetic analysis (Fig. 4b).

### Lineage-specific loss of antisense transcription

**Fig. 5:**
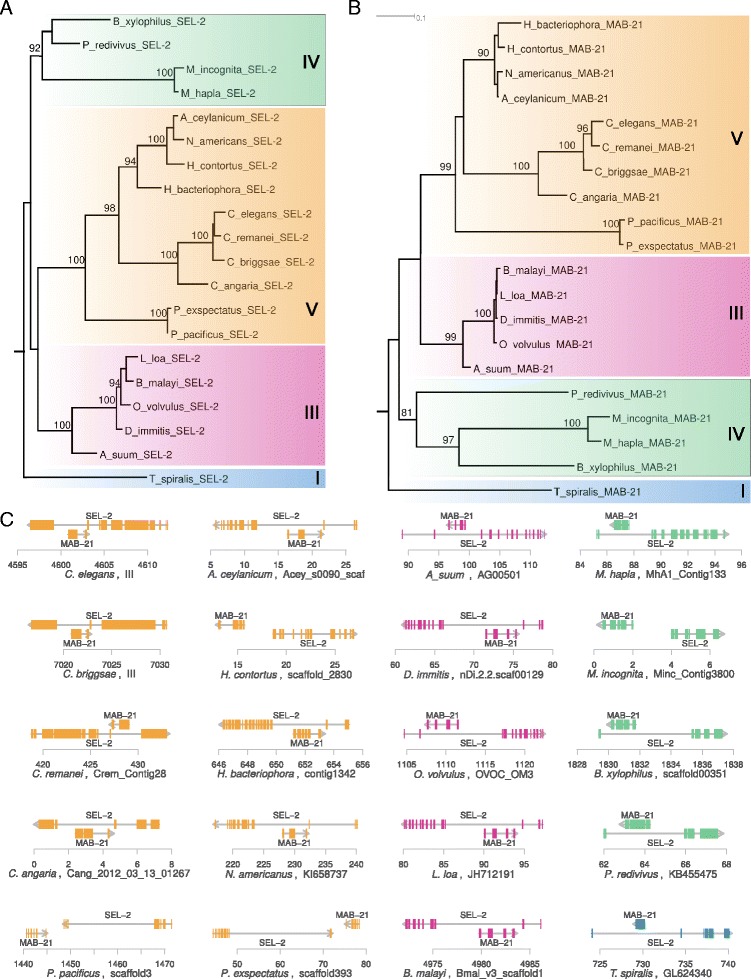
Lineage-specific loss of antisense transcription at the *sel-2* locus. **a** Gene tree of *sel-2*. **b** Gene tree of *mab-21*. **c** Genomic configuration of host gene and ASTs at the *sel-2* and *mab-21* locus in 20 nematode genomes. The first exons of *sel-2* in *M. incognita* and *H. contortus* could only be identified on different contigs indicating towards some minor assembly problem in this region. However, in both *Pristionchus* species, alignments for *sel-2* are rather complete and *mab-21* has clearly translocated out of the longest *sel-2* intron

In the last example for a detailed analysis of a highly conserved gene with AST, we examined the conservation of antisense transcription at the *sel-2* locus. *sel-2* has been shown to function as negative regulator of *lin-12/NOTCH* activity in vulval precursor cells (VPCs) of *C. elegans* [34]. The AST *mab-21* is involved in cell fate choice of cells in the tail of *C. elegans* males [35]. The phylogenetic trees of the host gene *sel-2* and *mab-21* show good congruence with the species tree, indicating that we have identified the orthologous regions in all twenty genomes (Fig. 5a–b). *mab-21* is embedded within the largest intron of *sel-2* in all nematode clades (Fig 5c). However, in some lineages, it seems as if gene structures do not overlap. While this can be attributed to assembly problems in some cases, the presence of *mab-21* outside the *sel-2* locus in two independently assembled and annotated *Pristionchus* genomes strongly indicates, that the antisense transcription was lost in the *Pristionchus* lineage and obviously is not essential for the function of both genes. Together, these examples demonstrate that even in the most highly conserved cases, ASTs undergo substantial lineage-specific turnover in terms of the gene structure, which is consistent with the little overall conservation across the nematode phylum (Fig. 2).

### Host genes delineate a highly conserved, but heterogenous gene class with long introns

The above mentioned examples show that ASTs can exhibit many different kinds of structural changes while being localized in large introns of their host genes. At the same time, the host gene is broadly conserved across the nematode phylum. The latter statement is supported by the finding that around 60 % of *C. elegans* host genes have one-to-one orthologs in *P. pacificus*, which is much more than compared to 29 % for all *C. elegans* genes (*P*<10^−16^, Fisher’s exact test) [26]. In strong contrast, only 16 % of *C. elegans* ASTs have one-to-one orthologs in *P. pacificus* (*P*<10^−16^, Fisher’s exact test). Despite their high degree of conservation, host genes represent a functionally heterogenous class of genes, as Gene Ontology term enrichment analysis only detects few and not highly-significant overrepresentations (ATP binding (GO:0005524) and larval development (GO:0002164), *P*<10^−3^, [36]). At the level of protein domains, we found some gene families with DNA binding domains such as CCCH-type (PF00642, *P*<10^−8^, Fisher’s exact test with FDR correction) and PHD-finger zinc fingers (PF00628, *P*<10^−6^), as well as BTB domain containing genes (PF00651, *P*<10^−8^), but also ATP synthases (PF00895, *P*<10^−6^), phosphotransferases (PF01636, *P*<10^−5^), deoxyribonucleases (PF03265, *P*<10^−5^) and amino acid permeases (PF00324, *P*<10^−5^).

**Fig. 6:**
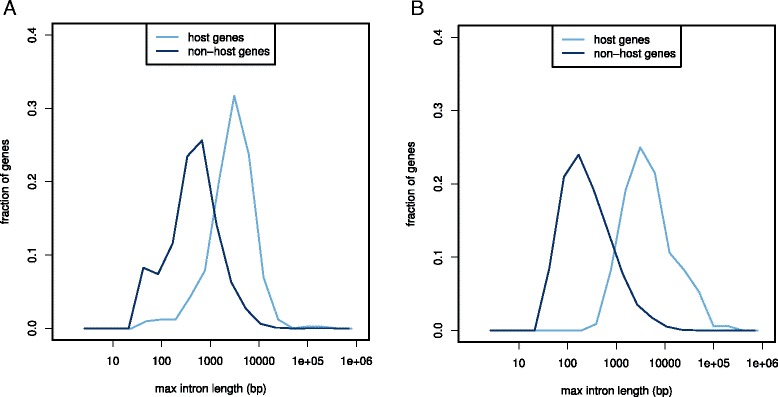
Long introns define host genes. **a** Distribution of intron lengths for host genes and non-host genes in *C. elegans* **b** Distribution of intron lengths in *P. pacificus*

Together with the finding that ASTs tend to reside in long introns of their host genes (Figs. 3c, 4c and 5c), we hypothesized that in general evolutionary constraint acts on preservation of these these long introns. However, since the size of a genomic region is proportional to the likelihood of being a target for translocation event (assuming uniformly distributed insertion probability across the whole genome), this makes long introns a more likely target for translocations of various genomic elements including other genes. The evolutionary conservation of long introns would also explain the unusually large genomic integrity of antisense gene pairs (Fig. 1g, h). To test for the evolutionary conservation of long introns in host genes, we compared the distributions of largest introns between host genes and non-host genes in *C. elegans* and *P. pacificus* (Fig. 6a, b). Indeed in both cases, the distribution of sizes of the largest intron per gene is strongly biased towards host genes having larger introns (*P*<10^−16^, Wilcoxon test).

### Little non-coding conservation between *P. pacificus* and *C. elegans*

The easiest explanation that would explain the conservation of large introns is the presence of important regulatory sequences. Such a setting would resemble the finding of genomic regulatory blocks in vertebrates that link regulatory elements with conserved synteny around developmental genes [37, 38]. To test whether introns of host genes harbor conserved regulatory elements, we performed BLASTN searches between non-coding/non-repetitive stretches of the *P. pacificus* and *C. elegans* genomes. In strong contrast to analyses in vertebrates that identified thousands of highly conserved non-coding elements between human and zebrafish [37, 39], we only found 28 non-coding and non-repetitive sequences in *P. pacificus* with recognizable homology at the nucleotide level to 27 sequences in *C. elegans* (BLASTN *e*-value <0.001). We thus speculate that long introns of host genes harbor regulatory elements, but these elements evolve faster than the protein products of their targets, which is in contrast to studies in vertebrates that show abundant conservation at non-coding and protein level [37, 39].

## Discussion

In the last couple of years an increasing number of studies have demonstrated evidence for important regulatory roles of antisense transcription in various organisms ([5, 6, 40] see [7] for review). To characterize antisense transcription in the nematode *Pristionchus pacificus*, we sequenced a strand-specific RNA-seq library and identified 1112 configurations of antisense transcription. Classifying these cases showed that the majority represents ASTs that are fully embedded in introns of their host genes. In contrast to the presumed regulatory role as suggested by studies in yeast, humans and plants that identified patterns of coexpression as well as mutual exclusiveness [5, 6, 40], *P. pacificus* ASTs preferentially encode proteins and their expression is completely uncorrelated to the expression of their host genes. This suggests that there is no obvious regulatory interaction between ASTs and host genes. As it is difficult to compare studies that were based on different data sets, and methods, we would like to point out that the only study by Sun et al. which employed a common protocol for multiple organisms demonstrated an exceptional role of antisense transcription in nematodes [41]. More precisely, Sun et al. found that compared to several vertebrates and insects, *C. elegans* had the lowest proportion of antisense gene pairs as a fraction of all genes [41] but unfortunaley they did not test for their regulatory function. Despite the lack of obvious regulatory interactions between ASTs and their host genes in *P. pacificus*, we found a few cases that showed patterns of broad conservation across the nematode phylum, with the gene pair *vps-4* and *npr-23* representing the only gene pair that is conserved in all twenty nematode genomes. This could be indicative of some other kind of constraint acting on preservation of such gene configurations. As it is not possible to distinguish constraint acting on the host gene alone from evolutionary constraint preserving the antisense configuration, it is extremely difficult to assess whether the observed conservation is greater than expected. We therefore chose to gain further insights into the evolution of antisense gene pairs by investigating the most highly conserved cases in greater detail. We found that ASTs can undergo a variety of structural changes such as gene loss, translocations, and intron gain. The small degree of conserved association between host genes and ASTs let us hypothesize that antisense transcription is a feature of highly conserved genes with long introns. This could potentially explain, why a recent study that screened for non-coding RNAs in *C. elegans* explicitly ignored the top 1 % of genes with longest introns [20]. Such introns do not harbor any highly conserved non-coding elements that are recognizable by nucleotide level sequence identity between *C. elegans* and *P. pacificus*, which is in contrast to vertebrates where highly conserved elements can be found between humans and zebrafish [39]. Similarly, in contrast to studies in vertebrates where conserved non-coding elements are strongly clustered around highly conserved developmental genes [39, 42], host genes in nematodes, despite their evolutionary conservation, represent a very heterogeneous class of genes as is indicated by the lack of strong signals in functional enrichment analyses. Finally, since functionally characterized antisense transcripts represent a diverse class of biological sequences [8–13], which as a whole is only poorly understood, our analysis provides first insights into the nature and evolution of antisense transcription in nematodes, showing that, at least in *P. pacificus*, transcriptomic signals for antisense transcription are mostly dominated by embedded protein-coding genes. However, we cannot exclude that there are non-coding regulatory antisense transcripts in our *P. pacificus* data set, of which we only generally characterized the most abundant class. Yet our study demonstrated that they by far do not represent the bulk of ASTs and more rigorous filtering techniques [20, 43] are probably needed to distill a candidate set of antisense non-coding RNAs. The most interesting question remains what imposes the conservation on long introns in nematodes. It is tempting to speculate that they harbor regulatory elements such as enhancers and silencers or small RNAs, but even if this was true, our analysis has shown that these non-coding regulatory elements evolve so fast that they are not recognizable by sequence homology between *C. elegans* and *P. pacificus*, which stands in contrast to the conservation of protein sequences of their respective host genes. This explanation would be in accordance with Davidson’s theory on regulatory network evolution stating that while mutations in pleiotropic genes likely affect all their functions throughout development and will probably have quite severe effects, mutations in individual cis-regulatory modules will only affect one particular phase of activity and can therefore more easily be tolerated [44]. However further analysis at much closer evolutionary distance [45–47] would be needed to identify and characterize such regulatory networks in further detail.

## Conclusions

Our analysis shows that antisense transcription is a feature of highly conserved genes with long introns and ASTs tend to be less conserved and protein-coding genes that do not show any regulatory effect on their respective host genes. The large size and little sequence conservation of long host gene introns makes them an ideal target for translocation of other genomic elements including protein-coding genes that eventually end up as ASTs. Our results may help to guide other studies in different taxonomic groups and help to gain an unbiased view of antisense transcription.

## Methods

### Worm cultures and RNA-seq experiment

The *P. pacificus* reference strain PS312 (California) was kept on 6-cm plates with nematode growth medium (NGM) agar and was fed with a lawn of *Escherichia coli* OP50 grown in 400 *μ*l L-Broth. Cultures were maintained at 20 ° C. Culture populations were allowed to grow until their food was exhausted, immediately after which the cultures were processed for sequencing. Five mixed-stage plates were washed with 40 ml M9, centrifuged immediately at 1300 g for 4 min, rinsed with 40 ml 0.9 % NaCl treated with 40 *μ*l ampicillin and 40 *μ*l chloramphenicol and shaken gently for 2 h, and finally concentrated into a pellet by centrifugation and immediately frozen in liquid nitrogen. NEBNext Ultradirectional RNA Library Kit (Cat # E7420L) was used to prepare libraries. RNA-Seq libraries were sequenced as 2 ×101-bp paired-end reads on an Illumina HiSeq 2000, yielding 30.6 million reads. Raw reads were submitted to the NCBI sequence read archive (accession SRX1609204).

### Identification of antisense transcripts in *P. pacificus* and *C. elegans*

Raw reads were aligned to the *P. pacificus* genome assembly (version Hybrid2) by means of the software TopHat (version v2.0.14) [48] with default settings. The *P. pacificus* Hybrid2 assembly represents and improved version of the Hybrid1 genome assembly [24] that was generated by merging scaffolds and filling gaps in the assembly based on PacBio sequencing data of the reference strain (PS312) and with the help of the software PBJelly2 and GapCloser of the SOAP suite [49, 50]. This reduced the number of scaffolds from 18,083 to 12,395 and increased the N50 value from 1.2 to 1.4 Mb. We then assembled 42,821 transcripts using the reference-guided transcriptome assembler cufflinks (version v2.0.1) [51]. ASTs were defined as assembled transcripts that completely fall into non-exonic regions of a transcript on the opposite strand. Coordinate overlaps between transcripts from both strands were done using bedtools (version 2.17.0) [52]. Figure 1b shows the distribution of various classes of overlaps.

To define ASTs in *C. elegans*, we relied on the available gene annotations from Wormbase (release WS140). This yielded 467 ASTs and 362 host genes. One-to-one orthology predictions between *C. elegans* and *P. pacificus* were generated by best-reciprocal BLASTP searches including inparalog filtering [19, 26]. To compare the degree of conservation of antisense gene pairs with blocks of conserved synteny between *C. elegans* and *P. pacificus*, we ran the software CYNTENATOR [53] with the following parameter settings: -gap –0.5 -coverage 1 -thr 3 -length 2, to compute a baseline distribution of conserved gene orders between both species.

### Enrichment test for protein-coding genes

To test for evidence of translation and coding potential in ASTs, we employed proteome data from two previous studies [24, 25] and *P. pacificus* gene predictions. We then tested, to what extent ASTs show BLASTX hits in the corresponding protein databases and compared these numbers to sets of randomly chosen transcripts with a similar size distribution, i.e. ASTs were divided based on their length into 20 sets holding each 5 % of ASTs. For each set that corresponded to a specific length range, a number of transcripts equivalent to the number of ASTs in the respective set was randomly chosen from all transcripts in the corresponding length range. The random sampling was repeated 1000 times to generate a baseline distribution of expected number of BLASTX hits. As our transcriptome data has directional information, we filtered out BLASTX hits that referred to the reverse strand of the query transcripts.

### Analysis of various expression profiles

We downloaded expression-profiling data throughout the development of *P. pacificus* [26]. For *C. elegans*, 30 expression profiles from different developmental stages including embryo, larvae, and adults [54] as well as 20 expression profiles of mutants and different environmental conditions were downloaded. These data sets were taken to test whether antisense gene pairs are more likely to show either correlated or anticorrelated expression patterns than randomly chosen gene pairs (Fig. 1e, f).

### Identification of highly conserved antisense gene pairs

To screen for conserved antisense gene pairs, we defined protein sequences of antisense gene pairs from *C. elegans* as queries for TBLASTN searches (version 2.2.28+) against 20 nematode genomes. These genomes were retrieved from WormBase and pristionchus.org: *Ancylostoma ceylanicum* (WS248), *Ascaris suum* (WS240), *Brugia malayi* (WS240), *Bursaphelenchelus xylophilus* (WS240), *Caenorhabditis angaria* (WS240), *Caenorhabditis briggsae* (WS240), *Caenorhabditis elegans* (WS240), *Caenorhabditis remanei* (WS240), *Dirofilaria immitis* (WS240), *Heterorhabditis bacteriophora* (WS240), *Haemonchus contortus* (WS240), *Loa loa* (WS240), *Meloidogyne hapla* (WS240), *M. incognita* (WS240), *Necator americanus* (WS248), *Onchocerca volvulus* (WS250), *Panagrellus redivivus* (WS240), *Trichinella spiralis* (WS240), *P. pacificus* (HYBRID2), and *P. exspectatus* (ALLPATH-LG) [55]. For each target contig and strand, leftmost and right most positions TBLASTN hits (*e*–value <0.001) for host gene and AST were determined and finally checked, whether the spanned region for the host gene hits contains the corresponding AST hits on the opposite strand (Fig. 2). To further confirm these results in individual cases, query sequences from *C. elegans* were mapped using exonerate (version 2.2.0) against the target genome (Figs. 3c, 4c and 5c). In individual cases we completed partial hits by using exonerate alignments of sequences with the help of more closely related species (e.g. taking the homologous regions of a closely related species as query instead of the *C. elegans* sequence).

### Phylogenetic analysis

Protein sequences for all 20 nematodes were extracted based on the exonerate alignments of the *C. elegans* queries. In a few cases protein sequences were manually completed using protein sequence data from available closely related species (WormBase, pristionchus.org, Genbank). We searched for paralogous genes and non-nematode orthologs in the NCBI nr database and generated multiple sequence alignments using MUSCLE (version 3.8.31) [56]. After model testing with the ProtTest webserver [57], the best model was then chosen to reconstruct a final Maximum-likelihood tree with bootstrap support values using the R package phangorn [58]. Orthologous sequences of non-nematode species (mostly from arthropods) were used to infer the root of individual trees. We searched for paralogs for given genes in the NCBI nr database but ignored near identical sequences, as it is difficult to decide whether these sequences represent different isoforms, assembly artifacts (e.g. different alleles were called as individual genes), or truly represent very recent duplications.

### Identification of highly conserved non-coding elements

Based on the repeat masked genome assemblies of *C. elegans* and *P. pacificus*, reciprocal BLASTN searches between non-coding and non-repetitve genomic regions were carried out. The repeatmasked *C. elegans* genome was downloaded from Wormbase (release WS240). For *P. pacificus*, repeats were modeled using RepeatModeler and masked using Repeatmasker. In both genomes, we masked any sequence that was annotated as protein-coding. The remaining regions with minimal length of 100 bp were then used as queries for BLASTN searches to find conserved non-coding elements, that we defined as BLASTN hits with *e*-value <0.001.

## References

[1] Zhang X, Zabinsky R, Teng Y, Cui M, Han M (2011). microRNAs play critical roles in the survival and recovery of *Caenorhabditis elegans* from starvation-induced L1 diapause. Proc Natl Acad Sci U S A.

[2] Spielmann M, Brancati F, Krawitz PM, Robinson PN, Ibrahim DM, Franke M, Hecht J, Lohan S, Dathe K, Nardone AM, Ferrari P, Landi A, Wittler L, Timmermann B, Chan D, Mennen U, Klopocki E, Mundlos S (2012). Homeotic arm-to-leg transformation associated with genomic rearrangements at the *Pitx1* locus. Am J Hum Genet.

[3] Ibrahim DM, Hansen P, Rödelsperger C, Stiege AC, Doelken SC, Horn D, Jäger M, Janetzki C, Krawitz P, Leschik G, Wagner F, Scheuer T, Schmidt-von Kegler M, Seemann P, Timmermann B, Robinson PN, Mundlos S, Hecht J (2013). Distinct global shifts in genomic binding profiles of limb malformation-associated *HOXD13* mutations. Genome Res.

[4] Kienle S, Sommer RJ (2013). Cryptic variation in vulva development by cis-regulatory evolution of a hairy-binding site. Nat Commun.

[5] Katayama S, Tomaru Y, Kasukawa T, Waki K, Nakanishi M, Nakamura M, Nishida H, Yap CC, Suzuki M, Kawai J, et al.Antisense transcription in the mammalian transcriptome. Science; 309(5740):1564–1566. 10.1126/science.1112009 16141073

[6] Havilio M, Levanon EY, Lerman G, Kupiec M, Eisenberg E (2005). Evidence for abundant transcription of non-coding regions in the *Saccharomyces cerevisiae* genome. BMC Genomics.

[7] Pelechano V, Steinmetz LM (2013). Gene regulation by antisense transcription. Nat Rev Genet.

[8] Tufarelli C, Stanley JAS, Garrick D, Sharpe JA, Ayyub H, Wood WG, Higgs DR (2003). Transcription of antisense RNA leading to gene silencing and methylation as a novel cause of human genetic disease. Nature Gen.

[9] Rinn JL, Kertesz M, Wang JK, Squazzo SL, Xu X, Brugmann SA, Goodnough H, Helms JA, Farnham PJ, Segal E, Chang HY (2007). Functional demarcation of active and silent chromatin domains in human HOX loci by non-coding RNAs. Cell.

[10] Swiezewski S, Liu F, Magusin A, Dean C. Cold-induced silencing by long antisense transcripts of an *Arabidopsis* polycomb target. Nature. 2009; 462. doi:10.1038/nature08618. 10.1038/nature08618 20010688

[11] Crampton N, Bonass WA, Kirkham J, Rivetti C, Thomson NH. Collision events between RNA polymerases in convergent transcription studied by atomic force microscopy. Nucleic Acids Res. 2006; 34. doi:10.1093/nar/gkl668. 10.1093/nar/gkl668 PMC1636470 17012275

[12] Beltran M, Puig I, Peña C, García JM, Álvarez A. B, Peña R, Bonilla F, García De Herreros A. A natural antisense transcript regulates Zeb2/Sip1 gene expression during Snail1-induced epithelial–mesenchymal transition. Genes & Development. 2008. doi:10.1101/gad.455708. 10.1101/gad.455708 PMC2275429 18347095

[13] Faghihi MA, Zhang M, Huang J, Modarresi F, Van Der Brug MP, Nalls MA, Cookson MR, St- G, Iii L, Wahlestedt C. Evidence for natural antisense transcript-mediated inhibition of microRNA function. Genome Biology. 2010; 11. doi:10.1186/gb-2010-11-5-r56. 10.1186/gb-2010-11-5-r56 PMC2898074 20507594

[14] Ragsdale EJ, Müller MR, Rödelsperger C, Sommer RJ (2013). A developmental switch coupled to the evolution of plasticity acts through a sulfatase. Cell.

[15] Mayer MG, Rödelsperger C, Witte H, Riebesell M, Sommer RJ (2015). The orphan gene dauerless regulates dauer development and intraspecific competition in nematodes by copy number variation. PLoS Genet.

[16] Rae R, Witte H, Rödelsperger C, Sommer RJ (2012). The importance of being regular: *Caenorhabditis elegans* and *Pristionchus pacificus* defecation mutants are hypersusceptible to bacterial pathogens. Int J Parasitol.

[17] Lightfoot JW, Chauhan VM, Aylott JW, Rödelsperger C (2016). Comparative transcriptomics of the nematode gut identifies global shifts in feeding mode and pathogen susceptibility. BMC Res Notes.

[18] McGaughran A, Rödelsperger C, Grimm DG, Meyer JM, Moreno E, Morgan K, Leaver M, Serobyan V, Rakitsch B, Borgwardt KM, Sommer RJ. Genomic profiles of diversification and genotype-phenotype association in island nematode lineages. Mol Biol Evol. 2016. doi:10.1093/molbev/msw093. 10.1093/molbev/msw093 27189551

[19] Baskaran P, Rödelsperger C (2015). Microevolution of duplications and deletions and their impact on gene expression in the nematode *Pristionchus pacificus*. PLoS One.

[20] Nam JW, Bartel DP (2012). Long noncoding RNAs in *C. elegans*,. Genome Res.

[21] Wang H, Chung PJ, Liu J, Jang IC, Kean MJ, Xu J, Chua NH (2014). Genome-wide identification of long noncoding natural antisense transcripts and their responses to light in *Arabidopsis*. Genome Res.

[22] Khorkova O, Myers AJ, Hsiao J, Wahlestedt C (2014). Natural antisense transcripts. Hum Mol Genet.

[23] Jan CH, Friedman RC, Ruby JG, Bartel DP (2011). Formation, regulation and evolution of *Caenorhabditis elegans* 3’UTRs. Nature.

[24] Borchert N, Dieterich C, Krug K, Schütz W, Jung S, Nordheim A, Sommer RJ, Macek B (2010). Proteogenomics of *Pristionchus pacificus* reveals distinct proteome structure of nematode models. Genome Res.

[25] Borchert N, Krug K, Gnad F, Sinha A, Sommer RJ, Macek B (2012). Phosphoproteome of *Pristionchus pacificus* provides insights into architecture of signaling networks in nematode models. Mol Cell Proteomics.

[26] Baskaran P, Rödelsperger C, Prabh N, Serobyan V, Markov GV, Hirsekorn A, Dieterich C (2015). Ancient gene duplications have shaped developmental stage-specific expression in *Pristionchus pacificus*. BMC Evol Biol.

[27] Djeddi A, Michelet X, Culetto E, Alberti A, Barois N, Legouis R (2012). Induction of autophagy in escrt mutants is an adaptive response for cell survival in *C. elegans*. J Cell Sci.

[28] Michelet X, Alberti A, Benkemoun L, Roudier N, Lefebvre C, Legouis R (2009). The ESCRT-III protein Cevps-32 is enriched in domains distinct from Cevps-27 and Cevps-23 at the endosomal membrane of epithelial cells. Biol Cell.

[29] Kim DW, Sung H, Shin D, Shen H, Ahnn J, Lee SK, Lee S (2011). Differential physiological roles of ESCRT complexes in *Caenorhabditis elegans*,. Mol Cells.

[30] Bargmann CI. Chemosensation in *C. elegans*. WormBook. 2006;:1–29. doi:10.1895/wormbook.1.123.1. 10.1895/wormbook.1.123.1 PMC4781564 18050433

[31] Blaxter ML, De Ley P, Garey JR, Liu LX, Scheldeman P, Vierstraete A, Vanfleteren JR, Mackey LY, Dorris M, Frisse LM, Vida JT, Thomas WK (1998). A molecular evolutionary framework for the phylum nematoda. Nature.

[32] Rödelsperger C, Streit A, Sommer RJ. Structure, function and evolution of the nematode genome. In: eLS. Chichester: John Wiley & Sons, Ltd.: 2013, doi:10.1002/9780470015902.a0024603.

[33] Chu J. S. -C, Chua SY, Wong K, Davison AM, Johnsen R, Baillie DL, Rose AM (2014). High-throughput capturing and characterization of mutations in essential genes of *Caenorhabditis elegans*. BMC Genomics.

[34] de Souza N, Vallier LG, Fares H, Greenwald I (2007). SEL-2, the *C. elegans* neurobeachin/lrba homolog, is a negative regulator of lin-12/Notch activity and affects endosomal traffic in polarized epithelial cells. Development.

[35] Mariani M, Baldessari D, Francisconi S, Viggiano L, Rocchi M, Zappavigna V, Malgaretti N, Consalez GG (1999). Two murine and human homologs of mab-21, a cell fate determination gene involved in *Caenorhabditis elegans* neural development. Hum Mol Genet.

[36] Jiao X, Sherman BT, Huang DW, Stephens R, Baseler MW, Lane HC, Lempicki RA (2012). DAVID-WS: a stateful web service to facilitate gene/protein list analysis. Bioinformatics.

[37] Kikuta H, Laplante M, Navratilova P, Komisarczuk AZ, Engström PG, Fredman D, Akalin A, Caccamo M, Sealy I, Howe K, Ghislain J, Pezeron G, Mourrain P, Ellingsen S, Oates AC, Thisse C, Thisse B, Foucher I, Adolf B, Geling A, Lenhard B, Becker TS (2007). Genomic regulatory blocks encompass multiple neighboring genes and maintain conserved synteny in vertebrates. Genome Res.

[38] Akalin A, Fredman D, Arner E, Dong X, Bryne JC, Suzuki H, Daub CO, Hayashizaki Y, Lenhard B (2009). Transcriptional features of genomic regulatory blocks. Genome Biol.

[39] Woolfe A, Goodson M, Goode DK, Snell P, McEwen GK, Vavouri T, Smith SF, North P, Callaway H, Kelly K, Walter K, Abnizova I, Gilks W, Edwards YJK, Cooke JE, Elgar G (2005). Highly conserved non-coding sequences are associated with vertebrate development. PLoS Biol.

[40] Wang XJ, Gaasterland T, Chua NH (2005). Genome-wide prediction and identification of cis-natural antisense transcripts in *Arabidopsis thaliana*. Genome Biol.

[41] Sun M, Hurst LD, Carmichael GG, Chen J (2006). Evidence for variation in abundance of antisense transcripts between multicellular animals but no relationship between antisense transcriptionand organismic complexity. Genome Res.

[42] Rödelsperger C, Köhler S, Schulz MH, Manke T, Bauer S, Robinson PN (2009). Short ultraconserved promoter regions delineate a class of preferentially expressed alternatively spliced transcripts. Genomics.

[43] Prabh N, Rödelsperger C (2016). Are orphan genes protein-coding, prediction artifacts, or non-coding RNAs?. BMC Bioinformatics.

[44] Erwin DH, Davidson EH (2009). The evolution of hierarchical gene regulatory networks. Nat Rev Genet.

[45] Prabhakar S, Poulin F, Shoukry M, Afzal V, Rubin EM, Couronne O, Pennacchio LA (2006). Close sequence comparisons are sufficient to identify human cis-regulatory elements. Genome Res.

[46] Katzman S, Kern AD, Bejerano G, Fewell G, Fulton L, Wilson RK, Salama SR, Haussler D (2007). Human genome ultraconserved elements are ultraselected. Science.

[47] Dillman AR, Macchietto M, Porter CF, Rogers A, Williams B, Antoshechkin I, Lee MM, Goodwin Z, Lu X, Lewis EE, Goodrich-Blair H, Stock SP, Adams BJ, Sternberg PW, Mortazavi A (2015). Comparative genomics of *Steinernema* reveals deeply conserved gene regulatory networks. Genome Biol.

[48] Langmead B, Trapnell C, Pop M, Salzberg SL (2009). Ultrafast and memory-efficient alignment of short DNA sequences to the human genome. Genome Biol.

[49] English AC, Richards S, Han Y, Wang M, Vee V, Qu J, Qin X, Muzny DM, Reid JG, Worley KC, Gibbs RA (2012). Mind the gap: upgrading genomes with pacific biosciences RS long-read sequencing technology. PLoS One.

[50] Li R, Zhu H, Ruan J, Qian W, Fang X, Shi Z, Li Y, Li S, Shan G, Kristiansen K, Li S, Yang H, Wang J, Wang J (2010). De novo assembly of human genomes with massively parallel short read sequencing. Genome Res.

[51] Trapnell C, Williams BA, Pertea G, Mortazavi A, Kwan G, van Baren MJ, Salzberg SL, Wold BJ, Pachter L (2010). Transcript assembly and quantification by RNA-seq reveals unannotated transcripts and isoform switching during cell differentiation. Nat Biotechnol.

[52] Quinlan AR, Hall IM (2010). BEDTools: a flexible suite of utilities for comparing genomic features. Bioinformatics.

[53] Rödelsperger C, Dieterich C (2010). Cyntenator: progressive gene order alignment of 17 vertebrate genomes. PLoS One.

[54] Gerstein MB, Lu ZJ, Van Nostrand EL, Cheng C, Arshinoff BI, Liu T, Yip KY, Robilotto R, Rechtsteiner A, Ikegami K, Alves P, Chateigner A, Perry M, Morris M, Auerbach RK, Feng X, Leng J, Vielle A, Niu W, Rhrissorrakrai K, Agarwal A, Alexander RP, Barber G, Brdlik CM, Brennan J, Brouillet JJ, Carr A, Cheung MS, Clawson H, Contrino S, Dannenberg LO, Dernburg AF, Desai A, Dick L, Dosé AC, Du J, Egelhofer T, Ercan S, Euskirchen G, Ewing B, Feingold EA, Gassmann R, Good PJ, Green P, Gullier F, Gutwein M, Guyer MS, Habegger L, Han T, Henikoff JG, Henz SR, Hinrichs A, Holster H, Hyman T, Iniguez AL, Janette J, Jensen M, Kato M, Kent WJ, Kephart E, Khivansara V, Khurana E, Kim JK, Kolasinska-Zwierz P, Lai EC, Latorre I, Leahey A, Lewis S, Lloyd P, Lochovsky L, Lowdon RF, Lubling Y, Lyne R, MacCoss M, Mackowiak SD, Mangone M, McKay S, Mecenas D, Merrihew G, Miller D. M. 3rd, Muroyama A, Murray JI, Ooi SL, Pham H, Phippen T, Preston EA, Rajewsky N, Rätsch G, Rosenbaum H, Rozowsky J, Rutherford K, Ruzanov P, Sarov M, Sasidharan R, Sboner A, Scheid P, Segal E, Shin H, Shou C, Slack FJ, Slightam C, Smith R, Spencer WC, Stinson EO, Taing S, Takasaki T, Vafeados D, Voronina K, Wang G, Washington NL, Whittle CM, Wu B, Yan KK, Zeller G, Zha Z, Zhong M, Zhou X, Ahringer J, Strome S, Gunsalus KC, Micklem G, Liu XS, Reinke V, Kim SK, Hillier LW, Henikoff S, Piano F, Snyder M, Stein L, Lieb JD, Waterston RH, m.N.C.O.D.E.C (2010). Integrative analysis of the *Caenorhabditis elegans* genome by the modencode project. Science.

[55] Rödelsperger C, Neher RA, Weller AM, Eberhardt G, Witte H, Mayer WE, Dieterich C, Sommer RJ (2015). Characterization of genetic diversity in the nematode *Pristionchus pacificus* from population-scale resequencing data. Genetics.

[56] Edgar RC (2004). MUSCLE: multiple sequence alignment with high accuracy and high throughput. Nucleic Acids Res.

[57] Darriba D, Taboada GL, Doallo R, Posada D (2011). ProtTest 3: fast selection of best-fit models of protein evolution. Bioinformatics.

[58] Schliep KP (2011). phangorn: phylogenetic analysis in R. Bioinformatics.

